# The Complement Anaphylatoxin C5a Induces Apoptosis in Adrenomedullary Cells during Experimental Sepsis

**DOI:** 10.1371/journal.pone.0002560

**Published:** 2008-07-02

**Authors:** Michael A. Flierl, Daniel Rittirsch, Anthony J. Chen, Brian A. Nadeau, Danielle E. Day, J. Vidya Sarma, Markus S. Huber-Lang, Peter A. Ward

**Affiliations:** 1 Department of Pathology, University of Michigan Medical School, Ann Arbor, Michigan, United States of America; 2 Department of Trauma, Hand and Reconstructive Surgery, University of Ulm Medical School, Ulm, Germany; Duke University Medical Center, United States of America

## Abstract

Sepsis remains a poorly understood, enigmatic disease. One of the cascades crucially involved in its pathogenesis is the complement system. Especially the anaphylatoxin C5a has been shown to have numerous harmful effects during sepsis. We have investigated the impact of high levels of C5a on the adrenal medulla following cecal ligation and puncture (CLP)-induced sepsis in rats as well as the role of C5a on catecholamine production from pheochromocytoma-derived PC12 cells. There was significant apoptosis of adrenal medulla cells in rats 24 hrs after CLP, as assessed by the TUNEL technique. These effects could be reversed by dual-blockade of the C5a receptors, C5aR and C5L2. When rats were subjected to CLP, levels of C5a and norepinephrine were found to be antipodal as a function of time. PC12 cell production of norepinephrine and dopamine was significantly blunted following exposure to recombinant rat C5a in a time-dependent and dose-dependent manner. This impaired production could be related to C5a-induced initiation of apoptosis as defined by binding of Annexin V and Propidium Iodine to PC12 cells. Collectively, we describe a C5a-dependent induction of apoptotic events in cells of adrenal medulla *in vivo* and pheochromocytoma PC12 cells *in vitro*. These data suggest that experimental sepsis induces apoptosis of adrenomedullary cells, which are responsible for the bulk of endogenous catecholamines. Septic shock may be linked to these events. Since blockade of both C5a receptors virtually abolished adrenomedullary apoptosis *in vivo*, C5aR and C5L2 become promising targets with implications on future complement-blocking strategies in the clinical setting of sepsis.

## Introduction

Septic shock accounts for an alarming number of deaths in intensive care units [1], [2] resulting in approximately 215,000 fatalities per year in the US alone, a number very similar to the numbers of deaths from acute myocardial infarction. More importantly, the mortality of patients with septic shock has increased over the last years [3] and septicemia ranks now as the 10^th^ leading cause of death in the U.S., as opposed to no. 13 in 1990 [4]. In fact, despite remarkable scientific and clinical advances, sepsis continues to be an incalculable and enigmatic disease for clinicians and researchers. Recent findings suggest that sepsis might not be a solitary, clearly defined disease, but rather a puzzling interplay of various biological systems and cascades with the immune system [5], resulting in a highly variable clinical picture, that we have yet to fully comprehend. One of the biological systems crucially involved in the pathogenesis of sepsis is the complement cascade. Especially, the complement anaphylatoxin C5a has been described to lead to adverse effects, when excessively generated during sepsis. Its detrimental part in the development of murine sepsis has been highlighted in a recent report [6]. Simultaneous double-blockade of C5a receptors (C5aR or C5L2) dramatically improved survival from 0% to 70–80%, stressing the focal position of C5a during experimental sepsis.

The brain and the immune system extensively communicate with each other during health and disease [7], [8]. To facilitate this correspondence, common “chemical languages” are employed. In addition to the hypothalamic-pituitary-adrenal (HPA) axis, the autonomic nervous system seems to be key in this “verbal exchange”, modulating inflammation via the adrenergic sympathetic and vagus-mediated, parasympathetic nervous systems [9], [10]. Both, endogenous catecholamines as well as vagus-derived acetylcholine seem to be part of this universal language, functioning as exquisite fine-tuners of the immune cells during inflammation [11]–[14]. Although catecholamines are frequently used last-resort drugs to stabilize cardiovascular functions during hemorrhagic shock and severe sepsis, their endogenous regulation during sepsis is poorly understood.

In the current study, we sought to evaluate the consequences of excessively generated C5a on the adrenal medulla, the body's major catecholamine-producing and storing organ, during sepsis. We hypothesized that, in line with all its reported adverse effects, excessive levels of C5a might greatly impair the adrenomedullary catecholamine production and/or their release during sepsis, which might result in septic shock.

## Results

### Experimental sepsis induces adrenomedullary apoptosis in vivo in a C5a-dependent fashion

**Figure 1:**
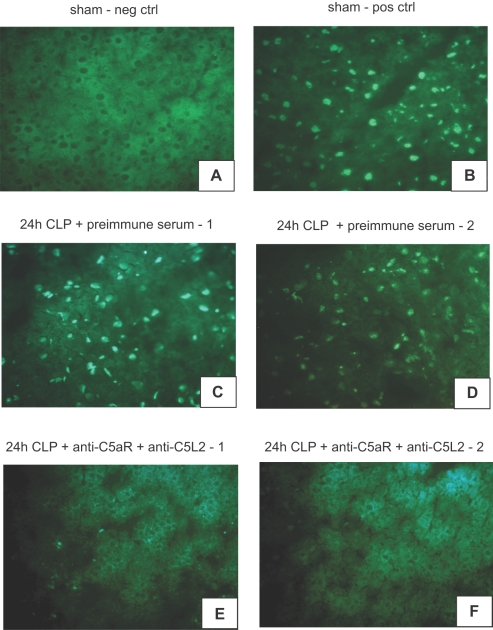
Induction of C5a dependent apoptosis in adrenal medullae after CLP. (A) Analysis of adrenal medulla in sham operated animals (neg ctrl) by the TUNEL technique. (B) Adrenal medullae of sham operated animals preincubated with DNAse before TUNEL staining served as positive control. (C, D) Examination of adrenal medullae obtained 24 hrs after CLP by TUNEL assay. (E, F) Adrenal medullae from animals with dual-blockade of C5aR and C5L2 acquired 24 hrs after CLP. Slides are representative of n≥3 animals per condition.

Animals were randomly assigned to 3 different groups: sham animals received abdominal midline incision and manipulation of the cecum (no ligation or puncture) and no treatment. Positive control rats received preimmune rabbit serum (5 ml i.p. 12 hrs before CLP) and were subsequently exposed to CLP. The third group of animals received anti-sera to C5aR and C5L2 12 hrs prior to CLP operation (1∶1 ratio, total of 5 ml, i.p.) followed by CLP. Twenty-four hrs after initiation of CLP, animals were euthanized, adrenal glands surgically removed and immediately embedded and frozen in OCT compound. Frozen sections (4 µm) were prepared from the embedded tissue and subjected to TUNEL analysis. As shown in Figure 1A, sham operated animals did not display adrenomedullary apoptosis and served as negative controls. Histological slides obtained from sham operated rats were preincubated with recombinant DNAse (10 min). The induced DNA strand breaks prior to TUNEL labeling served as positive controls (Figure 1B). When adrenal glands of preimmune rabbit serum treated rats were removed 24 hrs after CLP-induced sepsis and evaluated by TUNEL technique, there was significant apoptosis (Figure 1C, D). In sharp contrast, when both receptors for C5a were blocked by anti-sera to C5aR and C5L2, cells of the adrenal medulla from CLP rats failed to show significant signs of apoptosis (Figure 1E, F).

### CLP-induced sepsis is associated with antipodal levels of C5a and norepinephrine

**Figure 2:**
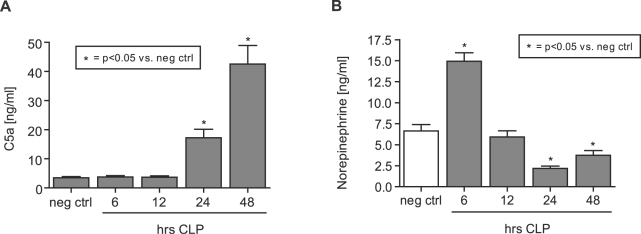
Antipodal levels of C5a and norepinephrine following CLP. Animals were subjected to CLP and plasma was obtained 0–48 hrs after initiation of experimental sepsis. (A) Plasma levels of C5a as a function of time following CLP. (B) Norepinephrine concentrations in plasma 0–48 hrs after CLP. All bars are presented as mean±s.e.m. For each bar n = 4–10.

Rats were subjected to cecal ligation and puncture (CLP)-induced sepsis, as previously described [15]. Plasma samples were obtained 0–48 hrs after CLP and analyzed for C5a and norepinephrine levels by ELISA. C5a levels were significantly elevated 24 hrs after initiation of experimental sepsis and peaked at 48 hrs (Figure 2A). In sharp contrast, there was a peak of plasma norepinephrine 6 hrs after initiation of CLP. Norepinephrine levels then fell below negative control levels 24 hrs and 48 hrs after CLP when compared to negative control levels (Figure 2B).

### Recombinant rat C5a decreases catecholamine-release from rat pheochromocytoma-derived PC12 cells

**Figure 3:**
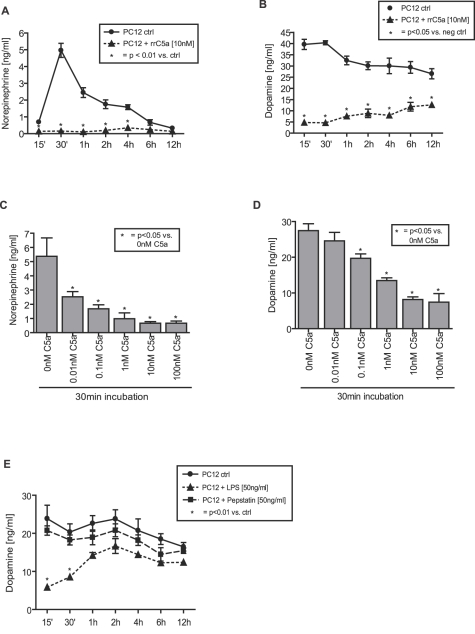
Impaired production of catecholamines by PC12 cells following C5a exposure. Norepinephrine levels in PC12 cell supernatants incubated with or without recombinant rat C5a (10 nM; rrC5a) as a function of time (A). PC12 cell supernatants incubated with or without 10 nM rrC5a subjected to dopamine analysis (B). Levels of norepinephrine (C) and dopamine (D) in PC12 cell supernatants following 30 min incubation with increasing doses of rrC5a (0 nM–100 nM). (E) Analysis of dopamine levels in PC12 cell supernatants after incubation with either LPS (50 ng/ml) or pepstatin (50 ng/ml) as a function of time. All bars are presented as mean±s.e.m. For each bar n = 5.

Rat PC12 cells derive from a catecholamine-producing neuroendocrine tumor of the adrenal medulla, termed a pheochromocytoma. They secrete norepinephrine and dopamine, but not epinephrine [16]. As shown in Figure 3A, cells not otherwise treated released norepinephrine, with levels peaking at 30 min and gradually declining thereafter. In contrast, incubation of PC12 cells with 10 nM recombinant rat C5a (rrC5a) completely abolished norepinephrine release over a long period of time (the entire 12 hr time span). Similar inhibitory effects were observed on dopamine release from pheochromocytoma cells (Figure 3B). The abridged secretion of norepinephrine and dopamine by PC12 cells following rrC5a exposure was dose dependent, reaching the inhibitory peak at a concentration of 10 nM rrC5a (Figure 3C, D). To assess if the presence of any non-specific protein had a similar effect on catecholamine release by PC12 cells, PC12 cells were incubated with pepstatin, a protein of similar size to rrC5a, as previously described [17]. Incubation of PC12 cells with pepstatin failed to significantly alter the time-course of dopamine release from pheochromocytoma PC12 cells, as demonstrated in Figure 3E. Moreover, exposure of PC12 cells to lipopolysaccharide (LPS, 50 ng/ml) led to only a transient decrease of dopamine release, returning to baseline levels after 2 hrs (Figure 3E), indicating that the long-term inhibition of catecholamine release by PC12 cells was unique to C5a. Since PC12 cells do not produce epinephrine [16], we were unable to evaluate its secretion.

### Exposure of PC12 pheochromocytoma cells to recombinant rat C5a induces apoptosis

**Figure 4:**
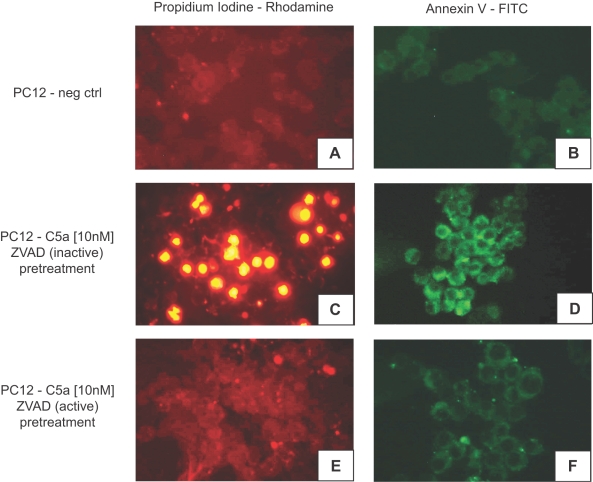
C5a-dependent induction of apoptosis in PC12 cells. (A, B) Incubation of PC12 cells in growth medium (30 min, 37°C) with subsequent analysis for apoptotic events by Propidium Iodine and Annexin V. (C, D) Preincubation of PC12 cells with the inactive derivate of the pan-caspase inhibitor ZVAD (1 hr, 37°C), followed by medium change, exposure to 10 nM rrC5a (30 min, 37°C) and staining for Propidium Iodine and Annexin V. (E, F) PC12 cells were pretreated with the active derivate of the pan-caspase inhibitor ZVAD (1 hr, 37°C), with subsequent medium change and incubation with 10 nM rrC5a (30 min, 37°C) and staining with Propidium Iodine and Annexin V. Staining was assessed using fluorescing microscopy and digital imaging. Slides are representative of n = 3 per condition.

To evaluate whether paralysis of catecholamine release from PC12 cells might be related to induction of apoptosis by rrC5a, PC12 cells were separated into three experimental groups: the negative control group was pretreated (1 hr, 37°C) and incubated (30 min, 37°C) in growth medium only. A positive control group received pretreatment (1 hr, 37°C) with the inactive derivative of the pan-caspase inhibitor ZVAD [Z-VAD-FMK(non-omethylated)] followed by medium change and subsequent incubation with 10 nM rrC5a (30 min, 37°C). A third group was pretreated for 1 hr at 37°C with the active derivative of the pan-caspase inhibitor ZVAD [Z-VAD(Ome)-FMK] followed by consecutive medium change and exposure 10 nM rrC5a (30 min, 37°C). Following incubation, PC12 cells were stained with Propidium Iodine or Annexin V. As depicted in Figure 4, negative control cells displayed virtually no staining for either Propidium Iodine or Annexin V (panels A, B). When cells were pretreated with inactive ZVAD and subsequently exposed to rrC5a (10 nM), there was a substantial fluorescence with Propidium Iodine or Annexin V stains (Figure 4C, D), indicating induction of apoptosis by rrC5a. These C5a-dependent effects were significantly reduced when PC12 cells were pretreated with the active derivate of the pan-caspase inhibitor, ZVAD (Figure 4E, F).

## Discussion

During sepsis, there is a robust activation of one of the phylogenetically oldest cascade systems of the body, the complement system [18]–[21]. Following activation via one of three different pathways, the complement cascade initiates the formation of the anaphylatoxins C3a and C5a, C4a and the terminal “membrane attack complex” (MAC). The complement system has been demonstrated to play a key role in the development of sepsis in rodents and humans [22], [23]. While physiological levels of complement activation products (such as C3a, C5a or MAC) might be beneficial and enhance bacterial clearance, excessive generation of C5a inflicts paralysis of phagocyte functions via paralysis of the MAPK-signaling pathways and inability to assemble the NADPH oxidase [24], [25], leading to abrogation of bactericidal functions: chemotaxis, phagocytosis and production of reactive oxygen species (“oxidative burst”). In addition to major disturbance of phagocyte function, excessive C5a levels during sepsis have been linked to impaired myocardial contractility and cardiac output [26], profound coagulatory and fibrinolytic changes [27], and enhanced production of proinflammatory cytokines and chemokines [17], [28], all of which result in reduced survival in rodents. Recently, the dual-blockade of both C5a-receptors, C5aR and C5L2, has been shown to greatly improve survival during moderate and severe experimental sepsis [6]. Thus, excessive complement activation with subsequent generation of C5a has been coined “too much of a good thing” [29] or “the dark side of C5a in sepsis” [30].

In the present study, we describe C5a-dependent induction of apoptosis of the adrenal medulla in septic rats (Figure 1) as well as C5a-induced apoptosis of pheochromocytoma-derived rat PC12 cells (Figures 3, 4). In line with our findings, a previous report described severe destructive ultrastructural lesions in the adrenal medulla and cortex during endotoxemia [31] and Tracey et al found adrenal medullary necrosis in the late phase of tumor necrosis factor (TNF)-induced shock in dogs [32]. Histologic examination of adrenal glands from calves, which succumbed to gram-negative septicaemia revealed thrombi, haemorrhage and necrotic changes [33].

During experimental sepsis, apoptosis seems to occur in many organs. Accordingly, apoptosis was noted in the thymus, spleen, Peyer's patches, liver, kidney, lung, intestine and skeletal muscle [34]–[38]. In line with these findings, septic humans present with focal apoptosis in the spleen, colon, and ileum [34], [39]. During sepsis syndrome, apoptosis of immune cells leads to a striking loss of lymphocytes and dendritric cells [40]–[42], which, in combination with the immunosuppressive effects of apoptotic cells, accounts for the profound immunoparalysis in the late course of sepsis. Autopsies of patients that died from sepsis have revealed extensive apoptosis of tissue lymphocytes and gastrointestinal epithelial cells [39], both cell types with high and rapid turnover. Administration of ZVAD, the same broad pan-caspase inhibitor used in the present study, improved survival during experimental sepsis and decreased lymphocyte apoptosis in a dose dependent manner in thymus and spleen [43].

Collectively, we present the first evidence for C5a-dependent apoptosis of pheochromocytoma PC12 cell and apoptosis of adrenal medulla cells triggered by excessive generation of C5a during experimental sepsis. Dual blockade of both receptors for C5a, C5aR and C5L2, during CLP-induced sepsis greatly ameliorated adrenomedullary apoptosis. Such events may result in inadequate levels of endogenous catecholamines in the circulation, perhaps being linked to the onset of septic shock. It remains to be determined if these findings are relevant to human sepsis and whether inhibition of adrenomedullary apoptosis might reduce the level of septic shock and improve the clinical outcome.

## Materials and Methods

### Animals and Anaesthesia

All investigative procedures and the animal facilities conformed to the Guide of Care and Use of Laboratory Animals published by the US National Institutes of Health. The study was approved by the University animal care and use committee (UCUCA) and performed according to appropriate guidelines. Specific pathogen-free male Long-Evans rats (300–325 g; Taconic, Hudson, NY), were anesthetized with isoflurane (Aerrane™, Henry Schein, NY, USA) and ketamine (100 mg/kg body weight) (Fort Dodge Animal Health, Fort Dodge, IA). Sepsis was induced by the CLP procedure as previously described [15]. In brief, after a midline incision, the cecum was exposed and ligated ∼1/2 of the distance from the distal pole. The ligated cecum was punctured through and through with a 19-gauge needle and a small portion of feces was expressed. The abdomen was closed in layers using 4–0 sutures (Ethicon Inc., Piscataway, NJ) and metallic clips.

### Preparation and characterization of antibody to mouse C5aR and C5L2

For the immunization of rabbits, we used mouse C5aR or C5L2 peptides (with the published sequences [44]) conjugated to keyhole limpet hemocyanin (Lampire Biological Laboratories). These antibodies to mouse C5aR and C5L2 have been shown to cross-react with rat C5aR and C5L2 [45], and have been demonstrated to be specific for each receptor and improve survival after CLP in mice [6].

Dual blockade of C5aR and C5L2 has been recently described [6]. Rats were intraperitoneally administered a single injection (5 ml) of mixed (1∶1) antiserum to C5aR (Lampire Biological Laboratories) and C5L2 (Lampire Biological Laboratories) or 5 ml of preimmune rabbit serum (control; Jackson Immunoresearch) 12 h before CLP.

### TUNEL assay

The terminal deoxynucleotidyl transferase dUTP nick-end labeling (TUNEL) technique was applied to determine the extent of adrenomedullary cell death in tissue sections using a Fluorescein in situ cell death detection kit (Roche Diagnostics, Indianapolis, IN) according to the manufacturer's instructions. Staining was assessed using fluorescing microscopy and digital imaging.

### Collection of plasma samples from septic animals

Blood was drawn by cardiac puncture and anticoagulated with citrate dextrose (ACD; ratio 9∶1). Blood samples were spun down at 2500 rpm for 10 min at 4°C. Plasma was collected and immediately frozen at −80°C until further analysis.

### ELISA analysis of rat C5a and norepinephrine

For determination of complement anaphylatoxin C5a levels in plasma samples of rats, an ELISA-system was developed. ELISA plates (Immulon 4HBX, Thermo Labsystems, Milford, MA, USA) were coated with purified polyclonal goat anti-rat C5a IgG (Invitrogen, Carlsbad, CA). This capture antibody is designed to recognize the C peptide region (amino residues 58–77) of rat C5a, as previously described [46]. After blocking of non-specific binding sites with 1% BSA (Sigma-Aldrich, St. Louis, MO) in PBS (Gibco-Invitrogen, Carlsbad, CA) containing 0.05% TWEEN 20 (Sigma-Aldrich, St. Louis, MO), the plate was coated with 100 µl diluted plasma and recombinant rat C5a [38] at defined concentrations for establishing the standard curve. As detection antibody, goat anti-rat C5a antibody (Invitrogen, Carlsbad, CA) was biotinylated (Pierce, Rockford, IL), and 100 µl/well were added. Following washing steps and incubation with streptavidin-peroxidase (R&D Systems, Minneapolis, MN), substrate (R&D Systems, Minneapolis, MN) was added and the color reaction was stopped with 1 N sulfuric acid. The absorbance was read at 450 nm.

For determination of plasma norepinephrine, samples were analyzed using a commercially available ELISA kit (Rocky Mountain Diagnostics, Colorado Springs, CO) according to the manufacturer's instructions.

### Preparation of rat recombinant C5a (rrC5a)

Preparation of rat recombinant C5a has been previously described [24].

### PC12 cell studies

Rat pheochromocytoma-derived PC12 cells were obtained from ATCC, (Manassas, VA). They were cultured according to the manufacturer's protocol. Cells were incubated as a function of time or dose-response with recombinant rat C5a. Supernatants were analyzed for norepinephrine or dopamine via ELISA kits obtained from Rocky Mountain Diagnostics (Colorado Springs, CO) according to the manufacturer's instructions.

### Fluorescing staining of PC12 cells with Annexin V and Propidium Iodine

PC12 cells were incubated with plain medium (neg ctrl) or medium containing rrC5a (10 nM) for 30 min, and assessed for Annexin V or Propidium Iodine binding with a commercially available Annexin V apoptosis detection kit (Santa Cruz, Santa Cruz, CA) according to the manufacturer's instructions. Staining was assessed using fluorescing microscopy and digital imaging. In a second set of experiments, PC12 cells were pretreated for 1 hr at 37°C with either plain medium, the pan-caspase inhibitor Z-VAD(Ome)-FMK (100 µM) or its inactive derivative (Z-VAD-FMK, 100 µM; both MP Biomedicals, Solon, OH). Following medium change, pretreated PC12 cells were incubated with 10 nM rrC5a (30 min at 37°C) and analyzed with the Annexin V apoptosis detection kit for Annexin V and Propidium Iodine as described above.

### Statistical analysis

All values are expressed as means±SEM. Data were analyzed with a one-way ANOVA and individual group means were then compared with a Student-Newman-Keuls test. Differences were considered significant when p≤0.05.
